# Differential correlates of fear and anxiety in salience perception: A behavioral and ERP study with adolescents

**DOI:** 10.3758/s13415-024-01159-y

**Published:** 2024-01-24

**Authors:** M. Oliveira, C. Fernandes, F. Barbosa, F. Ferreira-Santos

**Affiliations:** 1 Laboratory of Neuropsychophysiology, Faculty of Psychology and Education Sciences, University of Porto, R. Alfredo Allen, 4200-135 Porto, Portugal; 2 Faculty of Human and Social Sciences, University Fernando Pessoa, Porto, Portugal; 3 Research Center of IPO Porto (CI-IPOP, RISE@CI-IPOP (Health Research Network), Portuguese Oncology Institute of Porto (IPO Porto)/Porto Comprehensive Cancer Center (Porto.CCC), Molecular Oncology and Viral Pathology Group, Porto, Portugal

**Keywords:** Fear, Anxiety, Salience perception, Event-related potentials, Reaction times, Adolescents

## Abstract

Anxiety disorders are the most common psychopathologies among adolescents. Their diagnostic criteria include both fear and anxiety symptomatology, although according to the literature, we can find evidence for some distinction between these two emotions. The present study contribute to this distinction, exploring the effects of trait fear and trait anxiety on behavioral and neural correlates. Thirty-two participants (aged 11–16 years) performed two experimental tasks of salient target detection, including visual stimuli that were manipulated to become salient, while reaction times and EEG were recorded. Results of both tasks revealed differential effects of trait fear and trait anxiety assessed through the *Fear Survey Schedule for Children-Revised* and the *Youth Anxiety Measure for DSM-5* on reaction times and ERP components amplitudes. Specifically, higher symptoms from Separation Anxiety Disorder increased early neural visual processing and decreased reaction times for more salient stimuli. Also, trait fear reduced later neural visual processing of salient stimuli. These findings may provide a significant contribution to guiding psychological interventions, especially with adolescents presenting higher levels of anxiety-related symptomatology.

**Supplementary Information:**

The online version contains supplementary material available at 10.3758/s13415-024-01159-y.

## Introduction

Fear and anxiety frequently manifest in children and adolescents (Muris, 2011). In some cases, these emotions can surpass their functional and adaptive role becoming more intense and frequent, drifting into anxiety disorders—the most common psychopathologies among youth (Muris et al., 2017a, b; Narmandakh et al., 2021; Simon et al., 2017). Sharing “features of excessive fear and anxiety and related behavioral disturbances” (APA, 2013, p. 223), these disorders characterize fear and anxiety as two interconnected concepts (Grogans et al., 2023) that can assume a temporary manifestation—state, a more permanent manifestation—trait, or configure a psychopathological condition. Also, approaches to these constructs overlap at several levels, such as theoretical, functional, neurological, and symptomatologic (Sylvers et al., 2011). For instance, higher scores on both fear- and anxiety-related dimensions can be associated with emotion-related mechanisms on task, reflecting higher activations of the right hemisphere (Killgore & Yurgelun-Todd, 2007). Nevertheless, these two aversive emotions can be disentangled, as we can find in the literature evidence for (and against) this distinction (Daniel-Watanabe & Fletcher, 2021).

Across infancy and adolescence, fear experiences are usually mild and short, allowing the individual to quickly identify situations that can be a threat to survival. Therefore, fear can be characterized as being specific, present-focused, adaptative, and preparing the individual to react to an imminent (real or perceived) threat (Muris et al., 2017b). Anxiety does not have this purpose, causing hypervigilance even in the absence of a threat. Theoretically, anxiety relates to the “anticipation of future threat” (APA, 2013, p. 223), presenting a diffuse nature (Ohman, 2008) and causing significant suffering that, when extended in time, can lead to anxiety disorders. Fear is, therefore, an evolutionary response, leading the individual to “fight-flight-freeze” behaviors (Muris et al., 2017b; Ohman, 2008; Sylvers et al., 2011; Vriends et al., 2011) and protecting from something potentially harmful. Anxiety leads to attentional hypervigilance that consumes individual resources, often associated with emotional, cognitive, and physical repercussions (Macleod et al., 2019). Despite being a possible oversimplification, separating trait fear from trait anxiety can be useful for guiding research and clinical work. For example, higher levels of anxiety are associated in the literature with a cognitive bias leading individuals to detect threatening stimuli more rapidly (Adolphs, 2008). Furthermore, anxious people tend to maintain hypervigilance over time, delaying the disengagement from potential threats (Lee & Park, 2011; Whalen, 2007). However, these conclusions were not entirely corroborated by Kruijt and colleagues (2019) in their meta-analytical study of anxiety and selective attention, presenting arguments for and against this negative attentional bias.

The uncertainty or novelty of a situation can be interpreted as potentially threatening, eliciting more attentional resources due to prefrontal cortex activation, which assesses the real danger in the environment and facilitates learning (Whalen, 2007). Higher traits of anxiety can interfere with the frontal mechanisms that are essential in controlling attention and focus on a task, with a significant impact on top-down mechanisms (Pacheco-Unguetti et al., 2011). This deficit in prefrontal regulation hinders disengagement and deactivation from ambiguous situations, leading to increased processing of potentially threatening stimuli (Adolphs, 2008) and compromising individuals’ cognitive performance in relevant tasks. Moreover, some individuals maintain higher levels of activation even in the absence of a threat (Lee & Park, 2011).

Visual stimuli can signal a threat in the environment, becoming salient and acting as a cue that increases the significance of the stimuli (Adolphs et al., 1995). According to the Oxford Dictionary, salience refers to “the quality of being particularly important or easy to notice” (oxfordlearnersdictionaries.com). Thereby, visual salience may induce early visual processing because of its ambiguity (Holte & Phillips, 2009), novelty, unpredictability (Castro-Alamancos & Keller, 2011), or biological relevance (Tottenham et al., 2009). We also can increase the salience of visual targets by manipulating specific features that potentially increase the significance of the stimuli and the subject’s attention, facilitating target identification (Bordier et al., 2013). Salient targets seem to induce similar activation as biologically relevant stimuli (such as potential threats), modulating neural responses in task (Whalen, 2007). Several studies have investigated the effect of salience (namely visual salience) on human perception and behavior, although we find reasonable discrepancies amongst them regarding salience operationalization. For instance, some studies define visual salience based on the frequency or duration of eye gazes/fixations toward stimuli (Abeln et al., 2016; Sugano et al., 2013; Valuch et al., 2013), whereas others associate visual salience with the manipulation of visual features (Borji et al., 2013; Chen et al., 2013; Shen & Urminsky, 2013). Moreover, there are a few studies combining these two approaches to compute visual salience maps based on low visual features and eye movements (Fellrath & Ptak, 2015; Ma & Hang, 2015; Nakashima et al., 2015). Furthermore, a more behavioral approach through target detection and reaction times also is sustained, with salient stimuli being detected more quickly than nonsalient (Kompaniez-Dunigan et al., 2015; Mao et al., 2015; Penkunas & Coss, 2013). The present study included two tasks with different visual stimuli, namely circles and Gabor patches. Gabor patches are sinusoidal gratings, typically with a Gaussian envelope, that drive early visual activity (Male et al., 2020). The primary visual cortex is the first stage of visual cortical processing, where neurons are selective for simple stimulus attributes such as orientation. Therefore, the characteristics of these stimuli seem to match the special properties of V1 simple cells’ receptive fields, eliciting early components of visual evoked potentials (Baumgartner et al., 2017). In the Gabor patch, we can manipulate individually each feature, so that a stimulus can be deviant for that feature but standard for the others (Male et al., 2020). Despite the discrepancies in the operationalization of the concept, visual processing of salient stimuli can be assessed through event-related potentials (ERP) that are widely used to analyze brain activity time-locked to a specific event (Luck, 2014).

To assess immediate visual processing occurring in the early visual cortex, a positive component—*P100*—can be analyzed. This ERP component has an occipital topography that peaks in the 100–200-ms time window after the stimuli (Lee & Park, 2011). In addition, at approximately 250 ms after stimulus presentation, a negative component with parieto-occipital distribution can be observed, known as the *N200*. This component appears to be related to the assessment and discrimination of the visual properties of the stimuli (Olofsson & Polich, 2007; Olofsson et al., 2008), being partially modulated by top-down processes (Luck, 2014). ERP results suggest that the (eventual) cognitive bias in processing salient stimuli occurs at different stages, from earlier stages of neural processing and automatic allocation of attention to later stages where controlled cognitive processes occur (Lee & Park, 2011).

The present study aims to differentiate the effects of fear and anxiety-related symptomatology regarding trait fear and trait anxiety by analyzing behavioral and electrophysiological responses of adolescents to visual salience. Therefore, we analyzed neural correlates of the visual processing of salient stimuli in adolescents exhibiting different scores on psychopathological-related symptomatology, namely fear-related symptomatology (assessed through *The Fear Survey Schedule for Children–Revised;* Ollendick, 1983) and anxiety-related symptomatology (assessed through the *Youth Anxiety Measure for DSM-5;* Muris et al., 2017c). Trait fear and trait anxiety modulate perceptive processes, interacting with the responses to the stimuli with different levels of salience and different locations. We expect that these interactions have differential effects on earlier and later latency ERP components elicited by salient targets, as well as on reaction times (RT) regarding the response to target detection in the task.

To explore this, hypotheses for the present study were developed and are presented below:

- *H1*—The trait of Fear modulates perceptive processes, facilitating visual target discrimination and increasing *N200* amplitudes for salient stimuli;
- *H2*—Fear symptoms prepare the individual to react to imminent situations, reducing reaction times in salient target detection;
- *H3*—Higher trait anxiety symptoms enhance early visual processing indexed by *P100* for medium to high salient stimuli;
- *H4*—Higher scores of anxiety lead to faster behavioral responses in the detection of medium to high salient stimuli.

## Methods

### Participants

Our sample size was calculated by using G*Power 3.1 (Faul et al., 2007) considering the repeated measures ANOVAs with within-between interaction, including two groups and three levels of salience, with an effect size of *f* = 0.3, α = .05, and power = .95. The minimum sample size estimated was 32 participants (divided by the lower and higher scores’ groups).

The sample was nonprobabilistic and included 18 females and 14 males (9–16 years old; *M*_*age*_ = 12.6; *SD*_*age*_ = 1.7) who were enrolled in the present study, previously collected by convenience from a broader sample of 300 participants attending public schools from the North and Central regions of the country, previously assessed through an online protocol (Oliveira et al., 2023), including several instruments (e.g., *Fear Schedule Survey for Children-Revised* and *Youth Anxiety Measure for DSM-5).* These participants received an invitation to take part in the laboratory studies, along with a brief report on the protocol results. The sample for the present study represents 10.7% of the larger sample and was assembled after the guardian’s authorizations were provided by the participants. None of them presented formal psychiatric/neurologic diagnoses nor consumption of psychoactive medication. The study was favorably appraised by the local ethics committee and approved by the data protection unit.

### Self-Report measures

#### The Fear Survey Schedule for Children–Revised (*FSSC-R;* Ollendick, 1983; Dias & Gonçalves, 1999)

The *FSSC-R* includes 80 items assessing the intensity of five different fears (namely, *Fear of Failure and Criticism, Fear of the Unknown, Fear of Animals*, *Fear of Danger, Death, and Injuries*, and *Medical Fears*) in children and adolescents, through a three-point Likert scale (0 = *none;* 2 = *a lot*), also allowing to obtain a measure of global fear. Reliability analysis of the Portuguese version of *FSSC-R* revealed a very high Cronbach’s alpha (.96) for the total scale.

#### The Youth Anxiety Measure for DSM-5 (*YAM-5;* Muris et al., 2017c; Oliveira et al., 2023)

Part I of the Portuguese version of *YAM-5* includes 23 items and assesses symptoms of the main anxiety disorders rated on a four-point Likert scale (1 = *never;* 4 = *always*). This version revealed good internal consistency with McDonald’s Omega ranging from .68 to .88 across the four scales: *Separation Anxiety Disorder* (6 it.), *Selective Mutism* (4 it.), *Social and Generalized Anxiety Disorder* (9 it.), and *Panic Disorder* (4 it.), and for the total score of anxiety.

#### Pretask-related emotional state

Before starting the first task, participants self-reported their immediate emotional experience through a visual analog smiley-faces scale. The score ranged from 1 (green face) to 5 (red face). Higher scores reflected more discomfort, tension, or worry about performing the tasks because of it being a new and unknown experience. Although this score is not objectively related to a specific fear or anxiety experience, we included the pretask state measure in the fear dimensions for its specificity regarding the tasks.

### Experimental tasks

#### Task 1

**Fig. 1:**
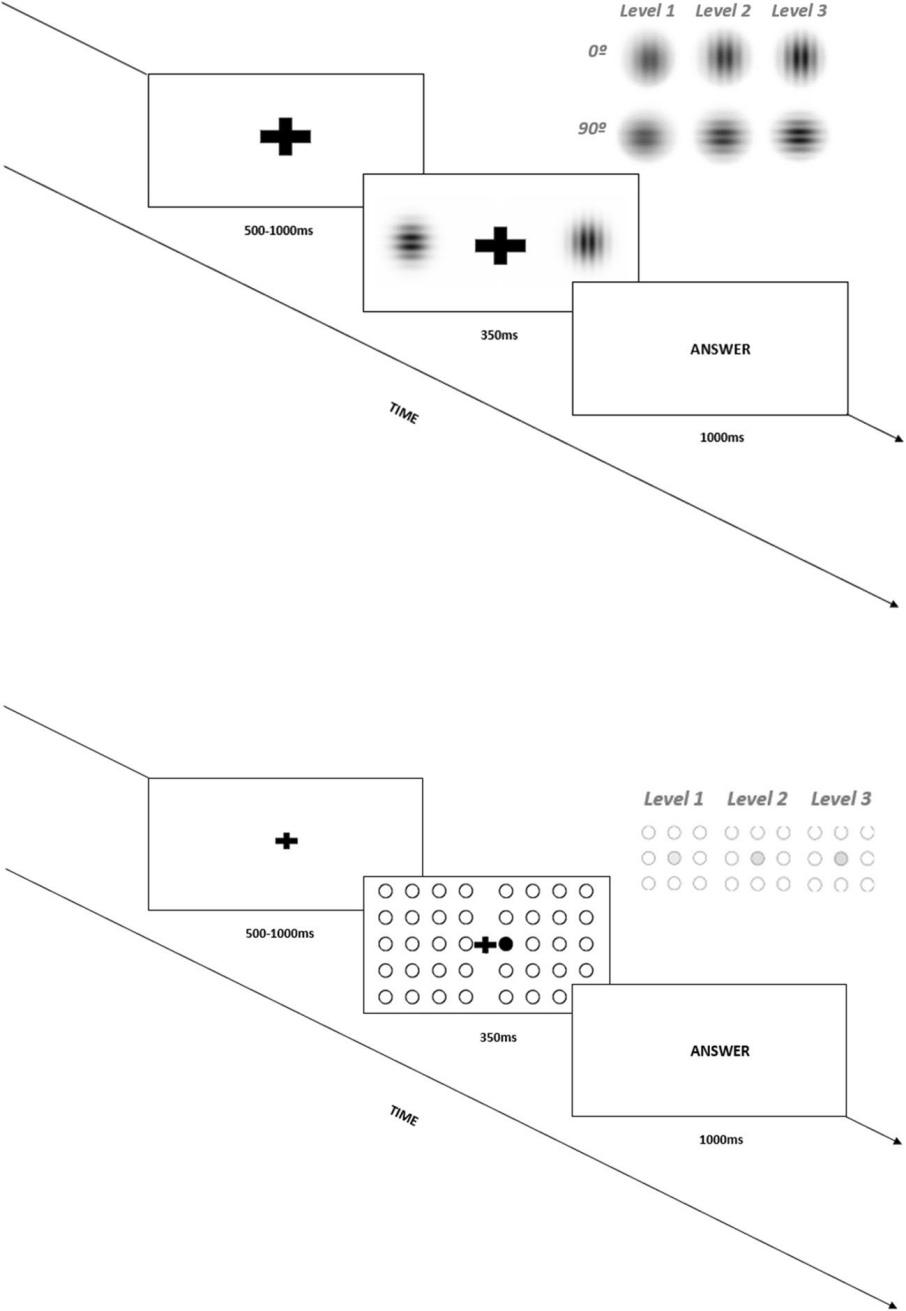
Experimental trial with level three of salience and the right location of the target, for Task 1 (above) and Task 2 (below)

A total of 280 images were displayed (40 trials *per* condition), including two Gabor Patches generated through an online patch generator (https://www.cogsci.nl/gabor-generator). The location and contrast of the salient stimuli were manipulated matching two locations and three different levels of salience (Fig. 1). Each image included a vertical patch (0° orientation) and a horizontal patch (90º orientation), both with the same level of salience. The vertical patch was defined as the target. Thereby, participants should identify the side (right/left) in which the vertical patch appeared on the screen. Visual salience was manipulated through three levels (see *Supplementary Material 1* for standard features and specific color features).

Before the task, participants received the instruction to press the right/left key on a 4-key response device corresponding to the location of the target (vertically oriented Gabor patch). The target had an equal probability of occurring on either side. To avoid motor-related EEG activity in the ERP components of interest, participants were instructed to wait for the slide displaying the word “answer” to respond, as quickly as possible, by pressing the correspondent key. Also, participants were instructed to not respond to nontarget stimuli (i.e., two horizontally oriented Gabor patches).

The task started with the instructions, which were followed by a training block of ten trials, and four experimental blocks of 70 stimuli separated by unlimited pauses, where participants were instructed to press any button to end the pause and start a new experimental block. The participants were also instructed to maintain fixation on a black cross in the center of the screen (to reduce saccades and head movements) while stimuli appeared in a random sequence with the vertically oriented Gabor patch emerging at one of the two equidistant locations surrounding the fixation cross. Gabor patch stimuli were presented during 350 ms since shorter periods of presentation could induce tiredness in the participants (Luck, 2014). The task was composed of 40 trials per condition to maximize the signal-to-noise ratio of our averaged ERP waveforms. With six experimental conditions and a neutral condition (without a salient target) to which participants should not respond, the stimuli for each condition were presented in a random order across participants, regarding two experimental factors: a) location of the salient target (right or left), and b) level of salience of the stimulus (levels *1*, *2*, or *3*). Each block included a ten times repetition for each condition. The experimental conditions were displayed unpredictably across the trial blocks to ensure that differences in ERPs across conditions were not a side effect of differences in arousal (Luck, 2014).

Both experiments presented a 2 x 3 factorial design (right/left location x three levels of salience). In both experiments, the participants attended to the right or left location according to the spatial localization of the salient stimulus. After each image was presented, the participant was allowed to respond to the “Answer” visual command by pressing a button (right or left). Each experimental run followed a 2350 ms (maximum) block design, and each image was preceded by a fixation cross presented randomly between 500 and 1000 ms duration, followed by the stimulus presentation of 350 ms and the answer slide with a maximum duration of 1000 ms, disappearing earlier with a button press.

#### Task 2

The *Task 2* replicated the experimental design of the *Task 1*. However, in this experiment, visual inputs were images composed of 40 circles (20 on each side, equidistant to the fixation point; Fig. 1). All circles were equal in size, white with black contours, except for one that was manipulated to become salient, exhibiting a grey instead of white color. The three levels of salience corresponded to three tones of grey: for the first level of salience, we created a 5% darker circle; for level 2, the circle was 15% darker than the others; and for level 3, the circle was 25% darker. The location of the salient circle was random on each side, with an equal probability of occurring on either side. This circle was defined as the target, and the participants were instructed to press the right/left key as quickly as possible during the response slide, according to the location of the target. Participants were instructed not to respond during nontarget stimuli (i.e., neutral condition composed of 100% white circles). Stimuli were presented for 350 ms, and subjects were instructed to maintain eye fixation on the central black cross while stimuli appeared in a random sequence regarding the level of salience and location of the target. As the property of color is processed very quickly (within approximately 50 to 100 ms; Hillyard & Anllo-Vento, 1998), the stimuli were likely to induce a bottom-up processing. This rapid extraction of information allows participants to react quickly (Pisella et al., 2000), for example, by pressing a button. Figure 1 includes the scheme of an experimental trial for each task, beginning with the presentation of a fixation cross, followed by the image, including the salient target, and finishing with the “answer” slide for motor response.

### Procedures

After collecting the informed consents from their guardians, the participant was seated at approximately 117 cm distance from the screen. The cap for EEG acquisition was then placed and impedances were measured and corrected for each electrode. The stimuli were presented on a 17” screen with a refresh rate of 60 Hz, at a visual angle of 7.58° x 9.62°. The participants were instructed to remain as quiet as possible, using the pauses of the tasks to produce the necessary movements to adjust their posture. Task 1 and Task 2 were performed sequentially. E-Prime 2.0 (2011, Psychology Software Tools, Inc., Sharpsburg, PA) was used to create the tasks and collect responses.

### EEG acquisition and processing

EEG was recorded at the scalp with a 128-channel HydroCel Geodesic Sensor Net (Eletrical Geodesics Inc., EGI, Eugene, EUA). The signal was amplified through a Net Amps 300 amplifier (EGI) and referenced online to Cz. Raw data were prepared for analysis through MATLAB (v. R2014b, The MathWorks, Inc) and EEGLAB toolbox (v. 13.6.5b; Delorme & Makeig, 2004). The signal processing began with a down sample of the signal from 500 Hz to 250 Hz and applied high-pass (0.1 Hz) and low-pass (30 Hz) filters. Bad channels were identified and rejected (approximately 10% per subject). Independent component analysis decomposition was performed, and independent components containing noise (specifically, eye blinks, saccades, and cardiac activity) were subtracted from the signal. After ICA decomposition, we interpolated the channels previously removed through spherical interpolation and the signal was re-referenced to the average of all electrodes. Epoch segmentation was applied to the signal, creating 1000-ms duration epochs: 200 ms before and 800 ms after stimulus presentation. Therefore, our baseline was set at 200 ms before the stimulus onset. Through visual inspection, the remaining epochs containing artifacts were rejected. Conditions were averaged and channel measures were precomputed.

### Electrophysiological measures

The ERP components were computed through EEGLAB using the onset of the images as time-locking points. Two ERP components—*P100* and *N200* were extracted. The *P100* component corresponds to the first positivity occurring after stimulus presentation. Peak amplitude was extracted over the clusters O1/O2 (averaged amplitude of the electrodes e76/e83/e84/e66/e70/e71) with a time window of 100 ms around the grand-average peak. For Task 1, the peak occurred at 172 ms (left hemisphere) and 164 ms (right), whereas for Task 2, the peak occurred at 160 ms (left and right hemisphere).

The peak amplitude of the *N200* was extracted over the clusters PO7/PO8 (electrodes e89/e90/e95/e64/e65/e69). For Task 1, time windows between 228 ms and 328 ms (left hemisphere) and 184 ms and 284 ms (right) around the grand-average peak were considered for analyses, whereas for Task 2, a time window from 168 ms to 268 ms over the left and right hemispheres was analyzed. These two sensorial components are usually task-related, with respective amplitudes varying according to the low-level features of the stimuli. *P100* indicates the first stage of the stimuli visual processing, whereas *N200* reflects the discrimination processes of the stimuli features.

**Fig. 2:**
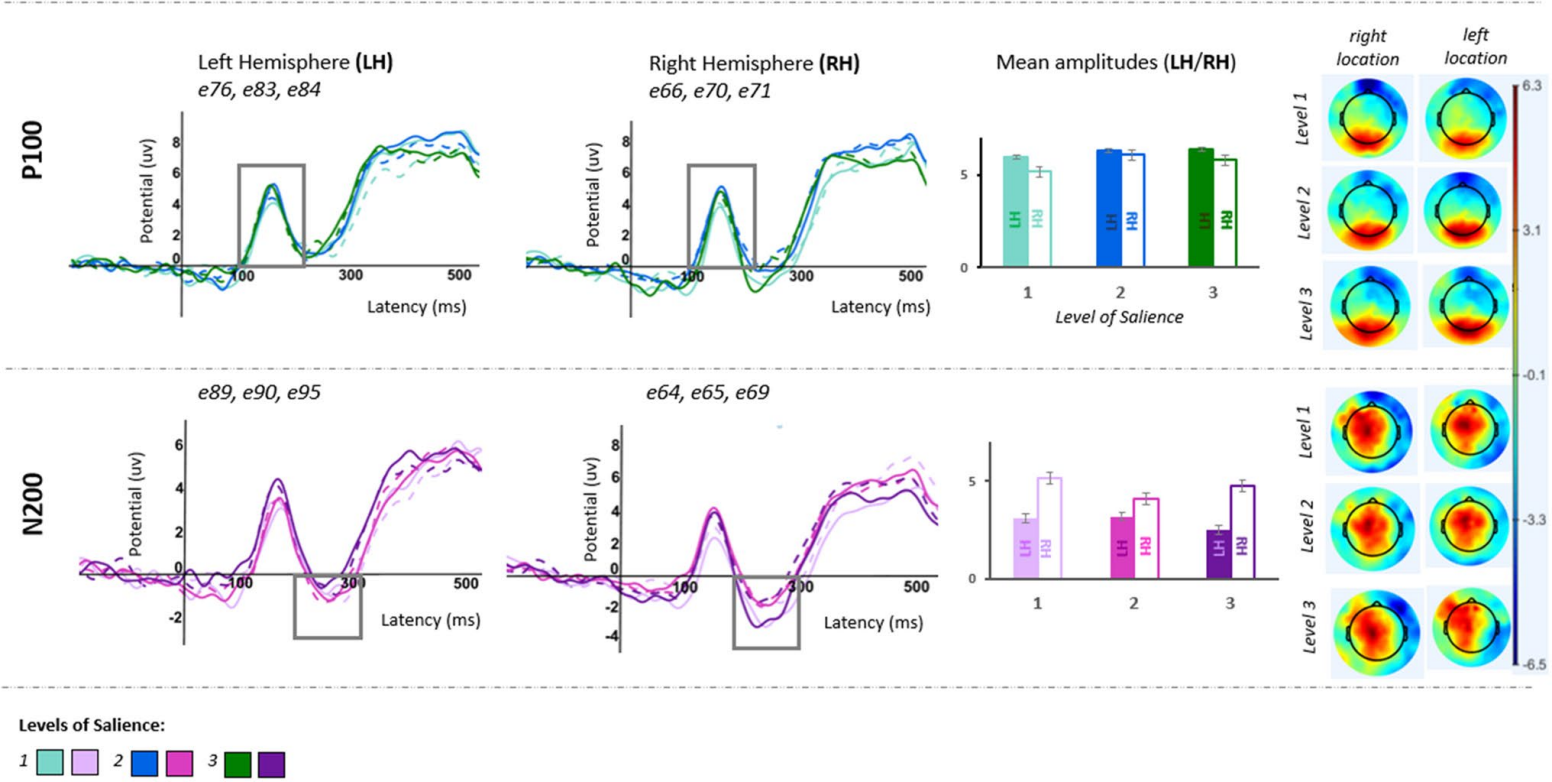
Task 1, ERP grand-averages illustrating P100 and N200 (by the level of salience in each hemisphere, dashed lines for right located targets), P100/N200 mean amplitudes (by the level of salience at right/left hemisphere), and topographic maps at P100/N200 time window (by the level of salience & location of the target) displaying the spatial distribution of brain electrical activity through the scalp in MicroVolts

**Fig. 3:**
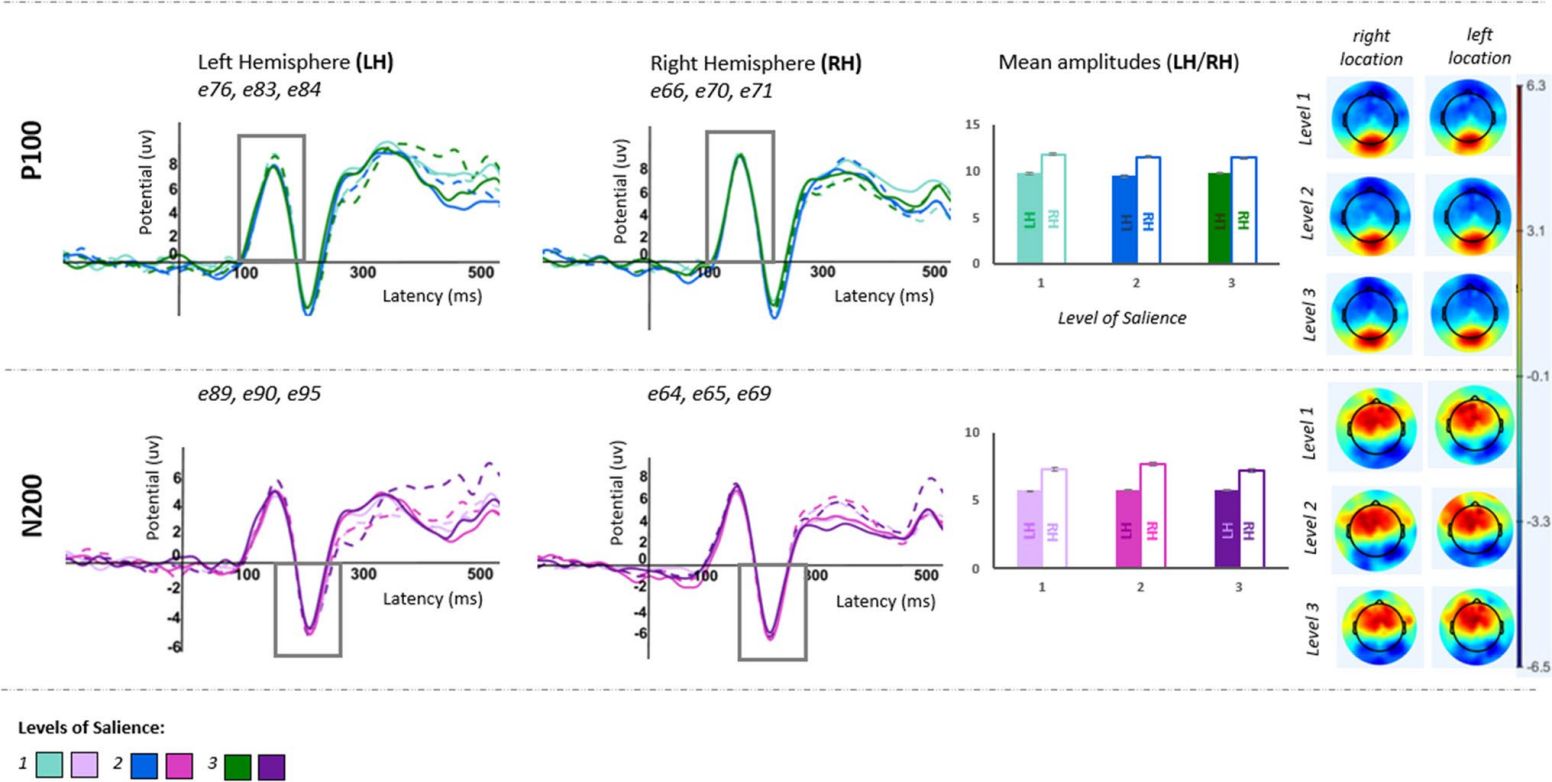
Task 2, ERP grand-averages illustrating P100 and N200 (by the level of salience in each hemisphere, dashed lines for right located targets), P100/N200 mean amplitudes (by the level of salience at right/left hemisphere), and topographic maps at P100/N200 time window (by the level of salience & location of the target) displaying the spatial distribution of brain electrical activity through the scalp in MicroVolts

The selection of time windows and regions of interest to measure each ERP component was data-driven (i.e., guided by the literature and based on the visual inspection of waveforms; Figs. 2 and 3).

### Statistical analyses

Statistical analyses were conducted on IBM-SPSS v.27. The dimensions assessed by *FSSC-R* and *YAM-5* scales were analyzed through descriptive statistics (*M, SD*) and the scales’ internal consistency (*McDonald’s Omega, Item-total correlations*), as well as intercorrelations.

To explore the effects of the dimensions of fear and anxiety on reaction times and ERP components of interest, we began to include each dimension as a covariate in ANCOVAs models. Our covariates included five scales of FSSSC-R* (Fear of Failure and Criticism, Fear of the Unknown, Fear of Animals*, *Fear of Danger, Death, and Injuries*, and *Medical Fears)* as fear dimensions and four scales of *YAM-5-I (Separation Anxiety Disorder*, *Selective Mutism*, *Social and Generalized Anxiety Disorder*, and *Panic Disorder*) as the anxiety dimensions. Also, the total scores of the scales were analyzed (global measures of fear and anxiety), as well as a measure of pretask emotional state. The interactions between each dimension and the within-subjects variables were analyzed, namely right/left hemisphere, right/left location, and levels 1/2/3 of salience (see *Supplementary Material* for complete report).

Repeated measures ANOVAs were conducted for behavioral and electrophysiological measures, with salience included as a within-subjects factor, and each dimension as a between-subjects factor. For that, a median split was performed to divide each dimension into two groups: one group with lower scores (LG) and the other with higher scores (HG). Reaction times were measured through E-Prime and averaged by condition. Whenever there was a violation of the assumption of sphericity, the Greenhouse-Geisser correction was applied. Differences between groups and levels of salience were explored through post hoc comparisons.

## Results

### Self-Report measures results

Dimensions of *FSSC-R and YAM-5-I* were assessed in the original sample of 300 participants (188 females; 11−16 years old; *M*_*age*_ = 13.1 years; *SD*_*age*_ = 1.3). The descriptive results, internal consistency coefficients, and intercorrelations are shown in Table 1.

**Table 1:** Descriptive statistics, internal consistency (ω) and correlation coefficients (r) for psychopathological dimensions (n = 300)

Psychopathological dimensions		*Mean*	*SD*	Item-total correlations	ω	*Pearson’s r*				
						*YAM-5-I*				
						SAD	SM	SGA	PD	*Total*
*FSSC-R*	Fear of failure and criticism	14.79	7.87	[.42; .66]	.91	.479	.187‡	.729	.494	.811
	Fear of the unknown	7.53	6.87	[.27; .69]	.89	.589	.199	.587	.490	.754
	Fear of animals	6.15	4.60	[.52; .63]	.86	.435	.127†	.400	.310	.526
	Fear of danger, death, and injuries	21.53	11.73	[.41; .74]	.94	.619	.137†	.522	.360	.664
	Medical fears	2.06	2.33	[.48; .74]	.80	.444	.115†	.431	.374	.622
	*Total*	52.17	28.86	[.21; .66]	.97	.630	.184‡	.649	.478	.779
*YAM-5-I*	Separation anxiety disorder	4.73	3.44	[.43; .67]	.77					
	Selective mutism	3.05	2.59	[.41; .52]	.68					
	Social/generalized anxiety disorder	10.01	5.72	[.48; .76]	.88					
	Panic disorder	2.16	2.67	[.68; .76]	.86					
	*Total*	19.95	10.78	[.14; .68]	.88					

*Notes:* ^†^*p* < .05; ^‡^*p* < .01; all other *p*-values < .001

All the psychopathological dimensions assessed evidenced good reliability and convergent validity, with high to very high internal consistencies (ω > .77), as well as significant intercorrelations, with most of them presenting medium to high values. Regarding the Selective Mutism scale from *YAM-5-I,* the correlation values with the other dimensions were low, as well as between the items of this scale and the total score of *YAM-5-I* (all the other items exhibited correlation values > .32 with total scale); the poorer psychometric properties of this scale were previously reported in other studies (Oliveira et al., 2023). In our sample, we achieved a coefficient omega (ω) > .85, with item-total correlations ranging from .29 to .82 regarding *FSSC-R.* Additionally, for *YAM-5-I,* ω > .70 was obtained for all scales and the total score (except for the Selective Mutism Scale, which presented ω = .57). Item-total correlations ranged between .30 and .89 for the scales.

### Behavioral and electrophysiological results

#### Task 1

Reaction times were collected for Task 1 regarding the correctly identified trials (mean accuracy rate of 72.3%). These trials were considered for ERP component analyses. Figure 2 summarizes the ERP components (*P100, N200*) for the three levels of salience (low/medium/high) and two target locations (right/left), in each hemisphere. A visual representation of each ERP component is provided for both the left and right hemispheres, corresponding to each level of salience and the right/left location of the salient target. Furthermore, the average amplitudes for each of the three salience levels in each hemisphere are displayed, along with topographic maps depicting brain activity during the ERP components’ time window by the level of salience/target location.

#### Task 2

Behavioral results revealed a mean accuracy rate of 94.2%. Correctly identified trials were considered for the analyses. ERP components (*P100*/*N200*) for the three levels of salience and two target locations, in each hemisphere, are illustrated in Fig. 3, as well as the average amplitudes for each of the three salience levels in each hemisphere and the topographic maps depicting brain activity during the ERP components’ time window by level of salience/target location.

### Effect of fear on salience perception

Fear dimensions were included as covariates in ANCOVA models, including hemisphere, localization, and salience, as within-subjects factors, to explore significant interactions between fear-related symptomatology, right/left hemisphere, right/left located targets, and levels 1/2/3 of salience (see *Supplementary Material 2* for the complete report). To analyze the impact of different levels of fear-related symptomatology in the neural processing of stimuli with different levels of salience, we focused specifically on the visual salience manipulation (three levels of salience, from less to more salient stimuli) and performed repeated measures ANOVAs with each psychopathological dimension included as a between-subjects factor (after a median split dividing the sample into two groups presenting lower and higher scores at that dimension), whereas salience was included as a within-subjects factor. A Power Analysis previously conducted ensured an adequate size of our sample for the subsequent analyses (*n* = 32).

#### Task 1

**Fig. 4:**
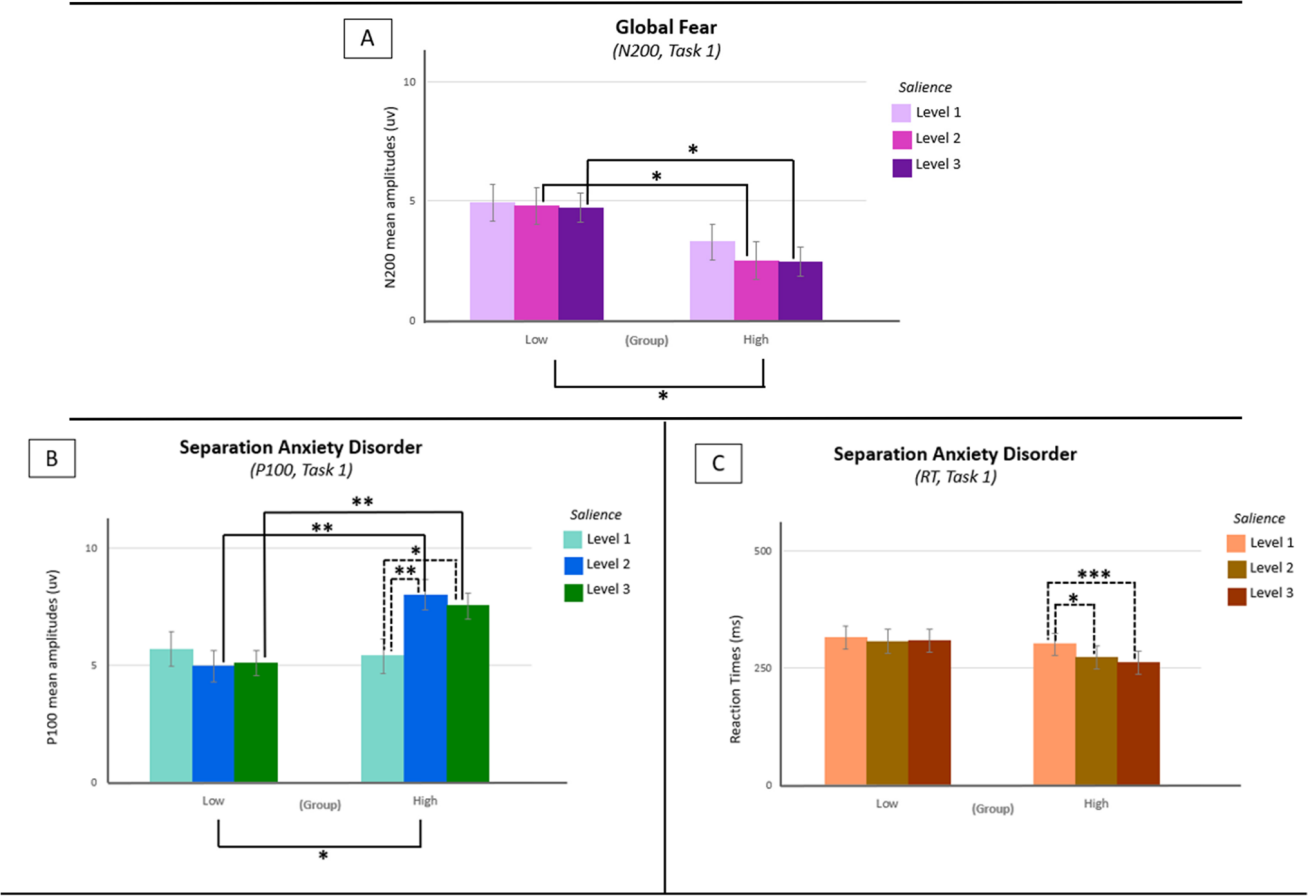
Tasks 1 & 2, group differences at P100/N200/RT by the level of salience, in fear and anxiety dimensions (dashed lines for within-group differences, error bars for SEMs)

Results did not evidence an effect of Fear dimensions on the early visual processing of salience, indexed by the *P100* component, or on the *Reaction Times*. However, later visual processing, indexed by the *N200* component, was significantly different between the lower and the higher scores group [*F*(1, 30) = 6.128, *p* = .019, η^2^_p_ = .170] regarding the *Global Fear* dimension (Fig. 4). Specifically, a main effect of salience was found at levels 2 [*F*(1, 31) = 4.821, *p* = .036] and 3 [*F*(1, 31) = 6.241, *p* = .018]. The group with lower scores exhibited increased *N200* amplitudes at levels 2 (*M* = −4.784; *SD* = 3.139) and 3 of salience (*M* = −4.711; *SD* = 2.483), as opposed to the higher scores group (Level 2: *M* = −2.495; *SD* = 2.746; Level 3: *M* = −2.460; *SD* = 2.613).

#### Task 2

Similar to Task 1, the *P100* component and the *Reaction Times* were not modulated by the fear dimensions in Task 2. However, a significant interaction was found between salience*Medical Fears when examining the *N200* component [*F*(2, 60) = 6.036, *p* = .004, η^2^_p_ = .168]. A closer look revealed differences between the lower scores group (*M* = −4.878; *SD* = 3.858) and the higher scores group (*M* = −8.311; *SD* = 3.004) at level 1 of salience [*F*(1, 30) = 7.730, *p* = .009], as well as significant differences in the group with lower scores regarding salience [*F*(2, 32) = 3.471, *p* = .043, η^2^_p_ = .178], although post-hoc comparisons did not confirm significant differences between each level of salience.

### Effect of anxiety on salience perception

Anxiety-related symptoms were also included as covariates in ANCOVA models, including hemisphere, localization, and salience (significant interactions described in *Supplementary Material 2).* Repeated measures ANOVAs were performed to assess how anxiety-related dimensions modulated neural and behavioral responses to visual salience in each task.

#### Task 1

We assessed the modulation of reaction times and ERP components of interest through anxiety-related symptomatology and found a significant interaction between salience*Separation Anxiety Disorder in the early visual processing, indexed by the *P100* component [*F*(2, 60) = 7.859, *p* < .001, η^2^_p_ = .208]. Specifically, differences between groups were found [*F*(1, 30) = 4.190, *p* = .049, η^2^_p_ = .123] with the higher scores group presenting significant differences in *P100* amplitudes between levels 1 (*M* = 5.386; *SD* = 0.881) and 2 (*M* = 8.015; *SD* = 0.786; *p* = .006) and 1 and 3 (*M* = 7.541; *SD* = 0.603; *p* = .016) of salience. Also, *P100* amplitudes were significantly different between groups at levels 2 [*F*(1, 31) = 8.960, *p* = .005; LG: *M* = 4.961; *SD* = 2.854; HG: *M* = 8.015; *SD* = 2.803) and 3 [*F*(1, 31) = 9.762, *p* = .004; LG: *M* = 5.097; *SD* = 2.402; HG: *M* = 7.541; *SD* = 1.776) of salience (Fig. 4). The *N200* component was not modulated by the Anxiety dimensions in this task, while a significant interaction between salience* Separation Anxiety Disorder was found when examining *Reaction Times* in task [*F*(2, 58) = 3.867, *p* = .027, η^2^_p_ = .118] (Fig. 4). Although there were no significant differences between groups (*F* < 1), we found differences between levels 1 (*M* = 300.776; *SD* = 30.814) and 2 (*M* = 272.666; *SD* = 31.580; *p* = .037), and between levels 1 and 3 (*M* = 261.790; *SD* = 31.704; *p* < .001) of salience, only for the higher scores group.

#### Task 2

No significant effects of anxiety dimensions were found on neural and behavioral responses to salience, in this task (*F* < 1). The three panels in Fig. 4 illustrate the results for significant effects of fear and anxiety in both tasks. Figure 4A illustrates the significant effects of the Global Fear dimension on *N200* amplitudes, in Task 1, with the lower scores group exhibiting significantly higher amplitudes of *N200* as opposed to the higher scores group (*p* < .05). Plus, post-hoc analyses found these differences occurring in levels 2 and 3 of salience. Figure 4B illustrates the main effect of the separation anxiety disorder dimension on *P100* amplitudes of Task 1, with significant differences occurring between LG and HG groups (*p* < .05), specifically in levels 2 and 3 of salience (*p* < .01), where the higher scores group also exhibited increased *P100* amplitudes. Plus, within this group, differences in *P100* amplitudes were observed between levels 1 and 2 (*p* < .01) and levels 1 and 3 (*p* < .05) of salience. Figure 4C illustrates the effects of the Separation Anxiety Disorder dimension on *Reaction Times* in Task 1, increasing the response times for level 1 of salience as opposed to levels 2 (*p* < .05) and 3 (*p* < .001).

## Discussion and conclusions

The distinction between fear and anxiety has been approached by the literature, with mixed evidence for a clear differentiation or a merging of these two concepts. Our study supports evidence for modulations of trait fear and trait anxiety in earlier and later visual processing of salient stimuli, as well as in reaction times.

Thirty-two adolescents reporting different levels of fear- and anxiety-related symptomatology performed two experimental tasks of salient target detection. EEG data were assessed and ERP components indexing perceptive processing were extracted, namely *P100* and *N200,* reflecting increased neural activity in response to salience. Reaction times also were extracted and averaged for each condition.

Globally, medium to high correlations between fear and anxiety dimensions were found. These results were expected due to an overlap of the traits of Anxiety and fearfulness already explored (Sylvers et al., 2011). However, evidence for a distinction between fear and anxiety also was approached earlier (Daniel-Watanabe & Fletcher, 2021), and the present work focused on making a valuable contribution to disentangling these two traits. Differential modulation was found for specific dimensions of fear and anxiety regarding reaction times and ERP components’ amplitudes.

Previous literature revealed inconsistent results regarding fear modulation of visual processing, namely due to methodological differences; for example, significant differences in *P100* amplitudes were found regarding fear recognition (Georgiou et al., 2018). In contrast, other studies reported greater amplitudes of *P100* regarding fear-related stimuli (Stefanou et al., 2019). In our study, we did not find evidence for significant differences in *P100* amplitudes between lower and higher scores groups regarding fear dimensions. However, global fear modulated later visual processing, indexed by *N200*, in Task 1. *N200* is associated with later visual processing (namely discrimination processes), which can be partially modulated by top-down processes (Luck, 2014), such as attention. For Task 1, *N200* amplitudes were higher for the group with lower scores on global fear, specifically for levels 2 and 3 of salience. For Task 2, differences between lower and higher scores groups regarding fear dimensions were not found. These results suggest that heightened fearfulness may impair the discrimination of visual features of more salient stimuli in later stages of visual processing, also modulated by top-down processes. Therefore, we rejected *H1* as trait fear decreases later visual processing of stimuli, namely for medium to high salient targets.

According to previous literature (Penkunas & Coss, 2013), we expected that heightened fearfulness reduced reaction times in target detection, as previous studies described shorter reaction times for the detection of potential threats (Bradley et al., 2005) to prepare the individual to react (Muris et al., 2017b), allowing a quick avoidance from threats (Soares et al., 2009). Nevertheless, these studies focused on fear evoked by the stimuli and not specifically a higher trait fear reported by the participants, as in the present study. In our study, we did not find significant differences between lower and higher trait fear regarding reaction times. Therefore, our second hypothesis was rejected.

Anxiety modulated neural and behavioral correlates in both groups with lower and higher scores on separation anxiety disorder for Task 1. Higher scores on this dimension enhanced early visual processing indexed by *P100* for levels 2 and 3 of salience, and for individuals with high levels of separation anxiety disorder, greater amplitudes of *P100* were found for levels 2 and 3 compared with level 1 of salience. Our third hypothesis stated that anxiety increased early visual processing of stimuli. Because larger amplitudes of *P100* were found when higher scores of anxiety were observed, but only for more salient stimuli, our *H3* was partially validated.

Finally, according to Adolphs (2008), more anxiety would lead to more rapid detection of targets because of a cognitive bias. Our results revealed reduced reaction times in detecting more salient targets compared with the less salient ones, only for the group with higher scores at the separation anxiety disorder dimension, whereas no differences were found between lower and higher scores groups regarding the anxiety-related dimensions. Our *H4* was, therefore, partially validated. The group with higher scores on separation anxiety disorder exhibited smaller reaction times only regarding levels 2 and 3 of salience compared with level 1. Therefore, a consistent modulation of electrophysiological and behavioral responses was found for the separation anxiety disorder dimension. Higher symptoms of separation anxiety disorder enhance early visual processing and shorten reaction times when detecting stimuli with higher levels of salience. We consider this as the main result of our study.

Despite the innovative nature of this research, which explored the distinct effect of trait fear and trait anxiety on the behavioral and neural processing of salient stimuli, the present study has limitations that can be addressed in future studies. First, although our two tasks were similar, they included different stimuli. Gabor patches in Task 1 evidenced more complexity than circles from Task 2, making it harder to discriminate between the vertical and the horizontal patch (explaining the higher accuracy rates found for Task 2). This discrimination difficulty may reduce visual processing at later stages, potentially affecting results in the task (Zanesco et al., 2019). Second, although a power analysis was conducted and the sample size was adequate for specific analysis, including salience as a within-subjects factor, the study could benefit from larger samples, potentially revealing more consistent results regarding ANCOVAS that included hemisphere and location as within-subjects variables. Finally, although our sample was divided into two groups with lower and higher scores for each dimension, there was not a clinical group with phobic versus anxious individuals; therefore, the results between the two groups cannot be generalized to clinical/nonclinical groups and must be interpreted with caution.

Nevertheless, a significant effort was made to disentangle fear and anxiety. Our main finding regarding the impact of separation anxiety disorder symptoms on early visual processing and reaction times can help clinicians working with adolescents to improve intervention programs specifically focused on anxiety symptomatology, promoting cognitive processing that will enable adolescents to interpret potential threats more accurately and, therefore, develop more effective coping strategies, instead of merely scanning the environment for these potential threats.

### Supplementary Information

Supplementary file 1 (DOCX 18.5 KB)

Supplementary file 2 (DOCX 30.4 KB)

## Data Availability

Data availability upon request.
