# Supplementary material for: Differential correlates of fear and anxiety in salience perception: A behavioral and ERP study with adolescents
Source: Cogn Affect Behav Neurosci. 2024 Jan 24;24(1):143–55. doi: 10.3758/s13415-024-01159-y (PMC10827851; doi:10.3758/s13415-024-01159-y)
Supplement: Supplementary file 1 — (DOCX 18.5 KB) [file 13415_2024_1159_MOESM1_ESM.docx]

**Parameters of Gabor patches generated through**

[**https://www.cogsci.nl/gabor-generator**](https://www.cogsci.nl/gabor-generator) **(Task 1)**

## Standard Features

#### **Size: 196 pixels**

#### **Envelope: Gaussian**

#### **Standard deviation: 12 pixels**

#### **Spatial frequency: 0.1 cycles/ pixel**

#### **Phase: 0 cycles**

#### **Background color: RED 255; GREEN 255; BLUE 255**

#### **Color 1: RED 100; GREEN 100; BLUE 100**

## Specific Features (salience manipulation)

#### **Color 2**

#### **Level 1: RED 80; GREEN 80; BLUE 80;**

#### **Level 2: RED 40; GREEN 40; BLUE 40;**

#### **Level 3: RED 0; GREEN 0; BLUE 0.**
