# Supplementary material for: Differential correlates of fear and anxiety in salience perception: A behavioral and ERP study with adolescents
Source: Cogn Affect Behav Neurosci. 2024 Jan 24;24(1):143–55. doi: 10.3758/s13415-024-01159-y (PMC10827851; doi:10.3758/s13415-024-01159-y)
Supplement: Supplementary file 2 — (DOCX 30.4 KB) [file 13415_2024_1159_MOESM2_ESM.docx]

**Report from ANCOVAs/ repeated measures ANOVAS including**

**hemisphere, localization, and salience as within-subjects factors,**

**and Fear and Anxiety dimensions as covariates**

The ANCOVA model for P100 and N200 included a 2x3x2 experimental design: 2 (target location: right, left) * 3 (levels of salience) * 2 (hemisphere: right, left), with location, salience, and hemisphere, as within-subjects variables. For reaction times, the experimental design was 2 (target location) * 3 (levels of salience).

## Effect of Fear

#### **Task 1**

Fear dimensions were included as covariates in ANCOVA models to assess the effects of fear-related symptomatology in the neural processing of salient stimuli. Exploring the effects of *Fear of Failure and Criticism* on the modulation of reaction times through the within-subjects variables, an ANCOVA was performed and a significant *Fear of Failure and Criticism*location*salience* interaction was found [*F*(2, 58) = 3.257, *p* = .046, η^2^_p_ = .101], with no significant differences in *location* or *level of salience*. To qualify this interaction, we analyzed separately two groups of participants obtained through a median split: a group with lower scores on the dimension *Fear of Failure and Criticism*, and another group with higher scores. This analysis allowed us to understand the direction of the effects. For the group with lower scores, results of the ANCOVA revealed differences in *salience* [*F*(2, 30) = 3.963, *p* = .041, η^2^_p_ = .209, ε = .801], although Pairwise Comparisons between the three levels of salience showed no significant differences (Bonferroni corrected). The results of the group with higher scores on this dimension revealed only a significant difference between levels 1 (*M* = 303.017; *SD* = 29.2) and 3 (*M* = 285.073; *SD* = 31.263; *p* = .031) of salience (lower salience, slower responses).

For the dimension *Fear of the Unknown*, results showed a significant interaction *Fear of the Unknown* location*salience* [*F*(2, 58) = 4.053, *p* = .023, η^2^_p_ = .123], with no significant differences in *location* (*F* <1) or *salience* regarding reaction times. Analyzing two groups separately, the group with lower scores did not present significant differences in *location, salience,* or their interaction, whereas the group with higher scores revealed significant differences between levels 1 (*M* = 286.625; *SD* = 25.109) and 3 (*M* = 258.250; *SD* = 26.986; *p=* .001) of *salience* [*F*(2, 28) = 5.341, *p* = .011, η^2^_p_ = .276]. The *Fear of Animals, Fear of Danger, Death, and Injuries,* and *Medical Fears* dimensions did not reveal significant interactions with *location, salience,* or their interaction. The global score of Fear (*total FSSC-R)* presented a significant *total FSSC-R*location*salience* interaction [*F*(2, 58) = 3.396, *p* = .040, η^2^_p_ = .105], although, in the groups with lower or higher scores, there were no significant interactions. The *Pre-task emotional state* was also analyzed as an instant measure of the emotional state of the participant; however, this score did not interact significantly with other within-subjects variables, regarding Reaction times in the task.

Results on the *P100* amplitudes when introducing each of the Fear dimensions into our ANCOVAs revealed no differences regarding *location* *salience*, *hemisphere*, or their interactions, for any of these dimensions.

Regarding *N200* amplitudes, results from the ANCOVAs revealed no significant effects of the dimensions *Fear of Failure and Criticism,* *Fear of the Unknown* in any of the within-subjects variables, or their respective interactions. The *Fear of Animals* presented significant *Fear of Animals*location*hemisphere* interaction [*F*(1, 30) = 6.301, *p* = .018, η^2^_p_ = .174]. For the group with higher scores, significant differences were found in *location* [*F*(1, 15) = 5.469, *p* = .034, η^2^_p_ = .267], *hemisphere* [*F*(1, 15) = 4.547, *p* = .050, η^2^_p_ = .233], and *location*hemisphere* [*F*(1, 15) = 8.294, *p* = .011, η^2^_p_ = .356], Pairwise Comparisons revealed greater amplitudes for left-sided targets (*M* = -4.0505; *SD* = .475) opposed to right-sided (*M* = -3.114; *SD* = .578 .034; *p =* .034), as well as at the right hemisphere (*M* = -4.389; *SD* = .745) opposed to the left (*M* = -2.775; *SD* = .460; *p =* .050), and also regarding the interaction, differences between location were significant only at the right hemisphere (*p =* .014). For the lower scores group, no significant differences were found. A significant interaction was found between the *location*hemisphere* Fear of Danger, Death, and Injuries* [*F*(1, 30) = 5.930, *p* = .021, η^2^_p_ = .165]. The group with lower scores on this dimension did not present a significant *location*hemisphere* interaction; however, a significant *salience*hemisphere* interaction [*F*(2, 30) = 3.608, *p* = .039, η^2^_p_ = .194) was found, although Pairwise Comparisons between the three levels of salience in each hemisphere showed no significant differences. For the group with higher scores on the *Fear of Danger, Death, and Injuries* dimension, significant differences were found on *hemisphere* [*F*(1, 15) = 5.266, *p* = .037, η^2^_p_ = .260), with greater amplitudes at the right (*M* = -4.651; *SD* = 0.914) opposed to the left (*M* = -2.482; *SE* = 0.378; *p = .037*) hemisphere, and on *location*hemisphere* [*F*(1, 15) = 16.361, *p* = .001, η^2^_p_ = .522), with higher amplitudes at the left (*M* = -5.533; *SD* = 0.879) opposed to the right (*M* = -3.768; *SD* = 1.081; *p =* .030) location of the target, only at the right hemisphere. Regarding the dimension of *Medical Fears,* there were no significant interactions with the other variables.

When *global Fear (total FSSC-R)* entered our ANCOVAs on *N200* amplitudes, a significant *total FSSC-R*location*hemisphere* interaction was found [*F*(1, 30) = 5.114, *p* = .031, η^2^_p_ = .146). The lower scores group on global Fear revealed a significant *salience*hemisphere* interaction [*F*(2, 30) = 4.953, *p* = .014, η^2^_p_ = .248), although Pairwise comparisons between each level of salience, at each hemisphere, were not significant. For the group with higher scores, the *location*hemisphere* interaction was found [*F*(1, 15) = 9.758, *p* = .007, η^2^_p_ = .394), with differences between left-sided location (*M* = -4.375; *SD* = 0.855) opposed to the right (*M* = -2.591; *SD* = 0.759; *p=* .025) only for the right hemisphere.

Finally, a significant *Pre-task emotional state*hemisphere*salience* interaction [*F*(2, 60) = 3.525, *p* = .036, η^2^_p_ = .105] was observed regarding N200 amplitudes. For the lower scores group, *location*hemisphere* [*F*(1, 18) = 5.963, *p* = .025, η^2^_p_ = .249] and *salience*hemisphere* interactions [*F*(2, 36) = 4.519, *p* = .018, η^2^_p_ = .201, ε = .824] were significant, although differences between *location* or *salience* in each hemisphere were not observed through Pairwise Comparisons. For the group with higher scores on this dimension, a main effect of *hemisphere* only was found [*F*(1, 12) = 10.491, *p* = .007, η^2^_p_ = .466], with greater amplitudes over the right hemisphere (*M* = -4.654; *SD* = 0.745) opposed to the left (*M* = -2.588; *SD* = 0.666; *p* = .007).

#### **Task 2**

The interactions between the Fear dimensions previously reported and other variables within-subjects were analyzed to explore Fear modulation on *Reaction times* and our ERP components of interest.

Regarding *reaction times*, there were no significant interactions between *Fear of Failure and Criticism, Fear of Animals, Fear of Danger, Death, and Injuries, Medical Fears, Pre-task emotional state,* and the other within-subjects variables. The dimension *Fear of the Unknown* presented a significant *Fear of the Unknown*location*salience* interaction [*F*(2, 58) = 4.053, *p* = .023, η^2^_p_ = .123), with a main effect of *salience* observed only for the group with higher scores on this dimension [*F*(2, 28) = 5.341, *p* = .011, η^2^_p_ = .276), presenting greater RT on level *1* (*M* = 268.624; *SD* = 25.109) compared to level *3* (*M* = 258.250; *SD* = 26.986; *p=* .001) of salience. Regarding the total score of FSSC-R, a significant *location*salience*FSSC-R* interaction was found [*F*(2, 58) = 3.396, *p* = .040, η^2^_p_ = .105), although no significant differences were found in the groups with lower and higher scores of global fear.

For *P100* amplitudes, we found significant interactions between Fear dimensions and other within-subjects variables. Specifically, a significant interaction regarding *Fear of Failure and Criticism***location*hemisphere* was found (*F*(1, 30) = 4.190, *p* = .049, η^2^_p_ = .123). The group with lower scores in this dimension exhibited a main effect of *hemisphere* (*F*(1, 16) = 9.593, *p* = .007, η^2^_p_ = .375), with greater amplitudes in the right (*M* = 10.927; *SD* = 1.651) opposed to the left (*M* = 9.013; *SD* = 1.435; *p* = .007) hemisphere. The higher scores group did not reveal significant differences between within-subjects variables. Regarding *Fear of Animals*, a significant interaction *Fear of Animals*location*hemisphere* [*F*(1, 30) = 4.905, *p* = .035, η^2^_p_ = .141] was observed. In the group with lower scores on this dimension, a main effect of *hemisphere* was found [*F*(1, 15) = 10.588, *p* = .005, η^2^_p_ = .414], with greater amplitudes at the right hemisphere (*M* = 10.666; *SD* = 1.717) opposed to the left (*M* = 8.664; *SD* = 1.476; *p=* .005), and no differences were found for the group with higher scores. Regarding the dimensions of *Fear of the Unknown*, *Fear of Danger, Death, and Injuries,* and *Medical Fears,* no significant interactions were found. Regarding the total score of *FSSC-R*, a significant *FSSC-R*location*hemisphere* interaction was found [*F*(1, 30) = 4.706, *p* = .038, η^2^_p_ = .136]. A main effect of *hemisphere* only was found for the lower scores group [*F*(1, 15) = 7.291, *p* = .016, η^2^_p_ = .327], with greater amplitudes at the right (*M* = 10.593; *SD* = 1.741) as opposed to the left (*M* = 8.871; *SD* = 1.527; *p* = .016) hemisphere. For the group with higher scores, no significant differences were found. A significant interaction was also obtained between *Pre-task emotional state* and the *hemisphere* (*F*(1, 30) = 4.770, *p* = .037, η^2^_p_ = .137); specifically, this main effect of *hemisphere* was found in the group with lower scores [*F*(1, 18) = 9.507, *p* = .006, η^2^_p_ = .346], with greater amplitudes observed in the right hemisphere (*M* = 12.955; *SD* = 2.042) opposed to the left (*M* = 9.958; *SD* = 1.458; *p* = .006). The group with higher scores on Pre-task emotional state did not present significant differences.

Regarding *N200* amplitudes, no significant interactions were found between *Fear of Failure and Criticism, Fear of Animals,* and other within-subjects variables. For the dimension *Fear of the Unknown,* a significant interaction *Fear of the Unknown*location*salience* was found (*F*(2, 60) = 3.890, *p* = .026, η^2^_p_ = .115), with the lower scores group presenting a main effect of *hemisphere* [*F*(1, 16) = 21.382, *p* < .001, η^2^_p_ = .572], with greater amplitudes observed in the right hemisphere (*M* = -7.076; *SD* = .817) opposed to the left (*M* = -4.743; *SD* = .579; *p* < .001). Also, a significant *location*salience* interaction [*F*(2, 32) = 3.311, *p* = .049, η^2^_p_ = .171] was observed in this group, with a significant difference between level 1 (*M* = -4.824; *SD* = .540) and level 2(*M* = -6.372; *SD* = .857; *p* = .033) of salience, only for left-sided targets. The higher scores group did not present significant differences. A significant interaction between *Fear of Danger, Death, and Injuries,* *location*, and *salience* was found (*F*(2, 60) = 4.326, *p* = .018, η^2^_p_ = .126). For the group with lower scores on this dimension, significant differences were found in *location* (*F*(1, 15) = 5.031, *p* = .040, η^2^_p_ = .251), *salience* (*F*(2, 30) = 3.610, *p* = .039, η^2^_p_ = .194) and *hemisphere* (*F*(1, 15) = 25.844, *p* < .001, η^2^_p_ = .633), with greater amplitudes regarding right-sided targets (*M* = -6.912; *SD* = .945) opposed to the left (*M* = -6.254; *SD* = .918; *p* = .040). Analyzing the differences between each level of salience through Pairwise Comparisons, no significance was found. Also, the right hemisphere revealed greater amplitudes (*M* = -7.719; *SD* = 1.093) opposed to the left hemisphere (*M* = -5.446; *SD* = 0.773; *p* < .001). The group with higher scores on *Fear of Danger, Death, and Injuries* did not reveal significant differences. For *Medical Fears,* a significant interaction *Medical Fears*salience* was observed [*F*(2, 60) = 7.571, *p* = .001, η^2^_p_ = .202)]. The group with lower scores on *Medical Fears* presented this main effect of *salience* [*F*(2, 32) = 3.473, *p* = .043, η^2^_p_ = .178)] but no significant differences were found in Pairwise comparisons, whereas the higher scores group presented a main effect of *hemisphere* [*F*(1, 14) = 5.554 *p* = .034, η^2^_p_ = .284)] with greater amplitudes elicited at the right hemisphere (*M* = -8.678; *SD* = .771) opposed to the left (*M* = -6.587; *SD* = .950). The total score of *FSSC-R* exhibited a significant interaction *FSSC-R*location*salience* [*F*(2, 60) = 4.106, *p* = .021, η^2^_p_ = .120), with the lower scores group presenting only a main effect of *hemisphere* [*F*(1, 15) = 18.051, *p* < .001, η^2^_p_ = .546], where greater amplitudes were observed at the right hemisphere (*M* = -7.919; *SD* = 1.137) opposed to the left (*M* = -5.506; *SD* = 0.778; *p* < .001). No significant differences were found in the higher scores group, for global fear. Regarding *pre-task emotional state*, no significant interactions were found between this dimension and the other within-subjects variables.

## 3.4 Effect of Anxiety

#### **Task 1**

To assess the modulation of RT and ERP components of interest through Anxiety-related symptomatology, Anxiety dimensions were included in ANCOVAs. Regarding *Separation Anxiety disorder, Selective Mutism,* *Social and Generalized Anxiety Disorder,* and *Panic Disorder,* we found no significant interactions of these dimensions with *location*, *salience,* or *location*salience* in reaction times*.* Significant interactions were found regarding the total score of YAM-5-I with significant *YAM-5-I** *location*salience* interaction [*F*(2, 58) = 3.491, *p* = .037, η^2^_p_ = .107]. Analyzing the two groups with lower and higher scores in this dimension, we found a main effect of *salience* for the group with lower scores [*F*(2, 30) = 4.240, *p* = .044, η^2^_p_ = .220, ε = .649] although Pairwise Comparisons did not reveal significant differences between levels of salience. No significant differences were found for the group with higher scores in this dimension.

For the *P100* we found a significant *Separation Anxiety disorder*salience* interaction [*F*(2, 60) = 3.246, *p* = .046, η^2^_p_ = .098], with a main effect of salience observed only for the group with higher scores in this dimension [*F*(2, 24) = 5.334, *p* = .012, η^2^_p_ = .308], with no significance between levels of salience in posterior comparisons. No other significant interactions were observed regarding the remaining Anxiety dimensions.

For the *N200*, we did not find significant interactions between *Separation Anxiety Disorder, Social and Generalized Anxiety Disorder, Panic Disorder,* and the total score of *YAM-5-I,* and the other within-subjects variables. We did find a significant interaction *Selective Mutism*location*salience* [*F*(2, 60) = 3.597, *p* = .033, η^2^_p_ = .107], with the lower scores group revealing a main effect of *hemisphere* [*F*(1, 16) = 5.398, *p* = .034, η^2^_p_ = .252] and *salience*hemisphere* interaction [*F*(2, 32) = 3.353, *p* = .048, η^2^_p_ = .173]. Greater amplitudes were observed in the right hemisphere (*M* = -5.064; *SD* = 0.876) as opposed to the left (*M* = -2.851; *SD* = 0.690; *p=* .034) in this group, although pairwise comparisons did not reveal significant differences between levels of salience in each hemisphere. For the group with higher scores, a main effect for *location* was found [*F*(1, 14) = 9.619, *p* = .008, η^2^_p_ = .407], with targets located at the left side eliciting higher amplitudes on N200 (*M* = -4.090; *SD* = 0.683) than right-sided (*M* = -3.051; *SD* = 0.678; *p=* .008).

#### **Task 2**

We examined how Anxiety dimensions modulated reaction times. Specifically, regarding *Separation Anxiety Disorder, Selective Mutism, Social and Generalized Anxiety Disorder,* and the total score of *YAM-5-I,* there were no significant interactions of these dimensions with the other variables. On the other hand, the *Panic* dimension revealed significant interactions with *location* [*F*(1, 30) = 5.584, *p* = .025, η^2^_p_ = .157] and *salience* [*F*(2, 60) = 3.103, *p* = .052, η^2^_p_ = .094]. Specifically, for the group with lower scores on this dimension, we found a main effect of *location* [*F*(1, 17) = 8.337, *p* = .010, η^2^_p_ = .329], with larger reaction times with left-sided targets (*M* = 295.595; *SD* = 21.905) opposed to the right-sided (*M* = 277.456; *SD* = 21.434; *p* = .010) For the group with higher scores, a main effect of *salience* was found [*F*(2, 26) = 3.840, *p* = .035, η^2^_p_ = .228], with significant differences between levels 1 (*M* = 344.619; *SD* = 34.696) and 3 (*p* = .028), and between levels 2 (*M* = 342.795; *SD* = 37.865) and 3 (*M* = 324.719; *SD* = 33.680; *p* = .022) of salience.

For the *P100* amplitudes, *Separation Anxiety Disorder, Social and Generalized Anxiety Disorder,* *Panic Disorder,* and the total score of *YAM-5-I,* did not have any significant interactions with our within-subjects variables. *Selective Mutism* presented a significant *Selective Mutism*salience* interaction [*F*(2, 60) = 3.978, *p* = .024, η^2^_p_ = .117], although in the group with lower scores of this dimension, a main effect of *hemisphere* was found [*F*(1,16) = 7.939, *p* = .012, η^2^_p_ = .332] with higher amplitudes at the right hemisphere (*M* = 13.507; *SD* = 2.172) opposed to the left (*M* = 10.448; *SD* = 1.527; *p* = .012). No significant differences occurred in the higher scores group.

Regarding *N200* amplitudes, *Separation Anxiety Disorder, Social and Generalized Anxiety Disorder,* and the total score of *YAM-5-I* did not present significant interactions with *location, salience, hemisphere,* or in-between interactions. A significant *Selective Mutism*salience*hemisphere* interaction was found [*F*(2,60) = 3.170, *p* = .049, η^2^_p_ = .096], with the group with higher scores on this dimension exhibiting a main effect of *hemisphere* [*F*(1,14) = 6.570, *p* = .023, η^2^_p_ = .319] specifically with differences between right (*M* = -7.265; *SD* = .765) and left hemisphere (*M* = -5.424; *SD* = .688; *p* = .023). Also, significant interactions were found between *Panic*location*salience* [*F*(2, 60) = 3.236, *p* = .046, η^2^_p_ = .097]. Analyzing the group with lower scores in this dimension, we found a significant *salience*hemisphere* interaction [*F*(2, 34) = 3.358, *p* = .047, η^2^_p_ = .165], with significant differences between levels 2 (*M* = -7.785; *SD* = 1.445) and 3 (*M* = -6.315; *SD* = 1.476) of salience, specifically in the right hemisphere. For the group with higher scores on the *Panic* dimension, no significant differences were found.
